# B Cell Adhesion to Fibroblast-Like Synoviocytes Is Up-Regulated by Tumor Necrosis Factor-Alpha via Expression of Human Vascular Cell Adhesion Molecule-1 Mediated by B Cell-Activating Factor

**DOI:** 10.3390/ijms22137166

**Published:** 2021-07-02

**Authors:** Sung-Sik Yoon, Eun-Yi Moon

**Affiliations:** Department of Bioscience and Biotechnology, Sejong University, 209, Neungdong-ro, Kwangjin-gu, Seoul 05006, Korea; yoonss6365@naver.com

**Keywords:** B cell adhesion, fibroblast-like synoviocytes, hBAFF, hVCAM1, TNF-α

## Abstract

Fibroblast-like synoviocytes (FLSs) play a key role in the pathogenesis of rheumatoid arthritis (RA) by producing inflammatory cytokines and interacting with various immune cells, which contribute to cartilage destruction. RA-FLSs activated by tumor necrosis factor alpha (TNF-α), exacerbate joint damage by triggering the expression of various inflammatory molecules, including human vascular cell adhesion molecule-1 (hVCAM1) and B cell-activating factor (hBAFF), with a role in maturation and maintenance of B cells. Here, we investigated whether B cell interaction with FLSs could be associated with hVCAM1 expression by TNF-α through hBAFF, using WiL2-NS B cells and MH7A synovial cells. TNF-α enhanced the expression of hVCAM1 and hBAFF. B cell adhesion to FLSs was increased by treatment with TNF-α or hBAFF protein. hVCAM expression was up-regulated by transcriptional activation of the hVCAM1 promoter(−1549 to −54) in MH7A cells treated with hBAFF protein or overexpressed with hBAFF gene. In contrast, hVCAM1 expression was down-regulated by treatment with hBAFF-siRNA. JNK was activated by TNF-α treatment. Then, hVCAM1 expression and B cell adhesion to FLSs were reduced by the treatment with JNK inhibitor SP600125. Transcriptional activity of hVCAM1 by the stimulation with TNF-α was inhibited by the deletion of −1549 to −229 from the hVCAM1 promoter. hVCAM1 expression and B cell adhesion to FLSs were reduced by treatment with hVCAM1-siRNA. Taken together, these results suggest that B cell adhesion to FLSs is associated with TNF-α-induced up-regulation of hVCAM1 expression via hBAFF expression. Thus, the pathological progression of RA may be associated with hVCAM1-mediated interaction of synovial cells with B lymphocytes.

## 1. Introduction

Rheumatoid arthritis (RA) is an inflammatory disease characterized by progressive joint destruction. Synovia exhibit an expanded neoangiogenesis, especially in hyperplastic tissues, facilitating an invasion of inflammatory cells [1]. Fibroblast-like synoviocytes (FLSs) are the predominant cell type in hyperplastic rheumatoid synovia. The hyperplastic tissue outgrows and infiltrates the cartilage and bone, causing joint destruction. In normal joints, the synovial lining comprises one to three cell layers; in RA, the number of cell layers may be as high as ten to 15 [2,3,4,5]. FLSs produce cytokines that perpetuate inflammation and proteases that contribute to cartilage destruction [2]. RA pathogenesis is accompanied by many immune cell types, including B and T cells and macrophages. The inflamed synovia contain numerous B cells [6,7]. B cells also produce chemokines, which promote leukocyte invasion into joints and lead to angiogenesis and synovial hyperplasia, which in turn cause chronic synovitis [8]. While interactions between B and T cells and between T cells and FLSs have been studied extensively [9,10], less attention has been paid to potential interactions between B cells and FLSs.

Human B cell-activating factor (hBAFF) is a member of the tumor necrosis factor (TNF) ligand superfamily and is also known as B lymphocyte stimulator or TNF superfamily member 13b. BAFF is a TNF homologue that controls apoptosis and mediates nuclear factor-jB, c-Jun N-terminal kinase (JNK), and TNF- and ApoL-related leukocyte-expressed ligand 1 [11]. BAFF expression is up-regulated by various cytokines, including TNF-α [12,13]. BAFF plays a crucial role in the homeostasis and maturation of B cells. Increased levels of BAFF are detected in the sera, synovial fluid, and saliva of RA patients. Furthermore, FLSs from RA patients (RA-FLSs) continuously express BAFF [14,15], and RA-FLSs stimulated by TNF-α exhibit significant increases in BAFF mRNA and protein levels [16]. However, little is known about BAFF-mediated molecules that regulate interactions between B cells and FLSs.

TNF-α is one of the major cytokines that mediate immunologic and pro-inflammatory processes in RA [17,18,19]. TNF-α is a member of the TNF ligand superfamily and is produced by both immune and non-immune cells [20]. A soluble form of TNF specifically binds to TNF receptor 1 (TNFR1), which is a type I transmembrane protein [21,22,23]. The binding of TNF to TNFR1 regulates inflammation, apoptosis, and cell proliferation and survival via the activation of intracellular signaling, including the nuclear factor-kappa B (NF-κB) and mitogen-activated kinase pathways [24,25]. TNFR1-induced signaling eventually induces the expression of target genes such as vascular cell adhesion molecule-1 (VCAM1) [24,25,26,27] and BAFF. However, it is unknown whether TNF-α regulates VCAM1 expression in FLSs through BAFF signaling.

VCAM1, identified in 1989, was initially named INCAM-110 and later was termed VCAM1 because of its ability to mediate firm melanoma cell adhesion [28,29]. VCAM1 belongs to the immunoglobulin (Ig) superfamily of proteins and consists of several extracellular Ig-like domains, a transmembrane region, and a cytoplasmic domain [28]. Generally, VCAM1 is expressed on the surface of endothelial cells under inflammatory conditions and mediates the rolling and adhesion of leukocytes and their recruitment from blood to tissue [14,30]. In 1990, the integrin α4β1 (also known as “very late activation antigen-4”, or VLA-4) was the first ligand of VCAM1 to be discovered [31]. VCAM1 can be cleaved from the endothelial surface to become soluble VCAM1, the level of which increases in the course of various diseases [32]. Due to its wide distribution in human tissues and organs, VCAM1 participates in numerous pathophysiological conditions, such as autoimmune diseases, infection, and cardiovascular disease [33,34,35]. During inflammation, VCAM1 on the surface of endothelial cells regulates leukocyte adhesion and transendothelial migration by interacting with α4β1 on the leukocytes [36]. VCAM1 molecules also contribute to B cell adhesion to FLSs [37]. However, it is unknown whether VCAM1 induced by TNF-α can regulate interactions between B cells and FLSs.

In this study, we investigated whether the interaction of B cells with FLSs is associated with TNF-α-induced human VCAM1 (hVCAM1) expression through BAFF signaling using WiL2-NS B cells and MH7A cells, a human RA synovial cell line. Our data suggest that the interaction of B cells with FLSs is associated with TNF-α-induced up-regulation of hVCAM1 expression via hBAFF expression. These results suggest that pathological progression of RA is associated with TNF-α-induced up-regulation of hVCAM1 expression and the interaction of synovial cells with B lymphocytes.

## 2. Results

### 2.1. MH7A Synovial Cells Interact with WiL2-NS B Lymphocytes

**Figure 1:**
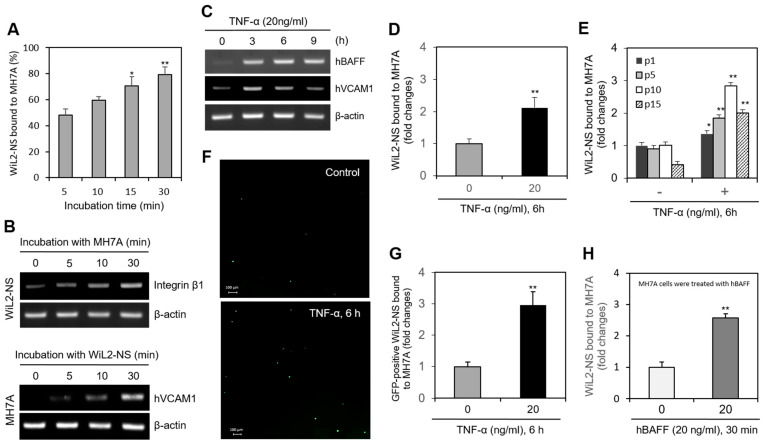
TNF-α enhanced a binding of B cells to synovial cells. (**A**,**B**) MH7A cells were plated overnight. Then, WiL2-NS cells were added and co-incubated to adhere for 5, 10, 15 and 30 min (**A**). Each cell was collected separately at each time point. RNA was purified with Nucleozol^®^. The expression of human vascular cell adhesion molecule-1 (hVCAM1) and integrin β1 in MH7A and WiL2-NS cells was respectively detected by RT-PCR (**B**). (**C**) Primary synoviocytes were treated with 20 ng/mL TNF-α for various times. RNA was purified with Nucleozol^®^. The expression of hBAFF and hVCAM1 was detected by RT-PCR. (**D**,**E**) MH7A cells were plated overnight and treated with 20 ng/mL TNF-α for 6 h. Then, WiL2-NS cells were added and co-incubated to adhere for 30 min. Unbound WiL2-NS B cells were washed out and MTT assay was used to analyze bound B cells (**D**). WiL2-NS cell adhesion to MH7A cells of each passage were measured by MTT assay (**E**). (**F**,**G**) WiL2-NS cells were transfected with pSG5-GFP plasmid DNA by using Viafect^®^. GFP-positive WiL2-NS cells were visualized under fluorescence microscope (**F**). Transfected WiL2-NS cells were co-incubated with MH7A cells at the ratio of 1:1 for 30 min. Unbound WiL2-NS B cells were washed out. Then, GFP-positive WiL2-NS cells were counted to analyze bound B cells (**G**). (**H**) MH7A cells were treated with hBAFF protein for 30 min. Then, WiL2-NS cells were added and co-incubated to adhere for 30 min. Unbound WiL2-NS B cells were washed out and MTT assay was used to analyze bound B cells. Each experiment was performed at least four times. Data in a bar graph represent the means ± SD. * *p* < 0.05, ** *p* < 0.01; significantly different from group with 5 min-incubation (**A**), TNF-α- (**D**,**E**,**G**) or hBAFF- (**H**) untreated control group.

We examined the interaction of FLSs and B cells using MH7A synovial cells and WiL2-NS B lymphocytes. When MH7A cells were incubated with WIL-2NS, the percentage of WiL2-NS cells bound to MH7A cells increased from 43% to 78% in a time-dependent manner (Figure 1A). Integrin β1 expression was enhanced in the WiL2-NS cells (Figure 1B, top), and hVCAM1 expression was enhanced in the MH7A cells (Figure 1B, bottom). As MH7A cells were co-incubated with WiL2-NS cells for up to 6 h, the expression of pro-inflammatory cytokine such as IL-6 was increased in MH7A cells significantly (Supplementary Figure S2). When the MH7A cells were treated with TNF-α for 3, 6, and 9 h, hVCAM1 expression steadily declined after 3 h, while hBAFF expression increased (Figure 1C). A 6-h treatment with TNF-α doubled the percentage of WiL2-NS cells bound to MH7A cells (Figure 1D). The percentage of WiL2-NS cells bound to MH7A cells also depended on the passage number (the number of times the cell line had been subcultured) of the MH7A cells. At the first, fifth, tenth, and fifteenth passage, MH7A synovial cells treated with TNF-α were bound to WiL2-NS cells with 1.3, 1.8, 2.8, and 4.0 times the affinity of the TNF-α-untreated control cells at each passage (Figure 1E). WiL2-NS cells transfected with the pSG5 plasmid containing a green fluorescent protein reporter gene were bound to TNF-α-treated (20 ng/mL, 6 h) MH7A cells with 2.9 times the affinity of the non-TNF-α-treated control cells (Figure 1F,G). MH7A and WiL2-NS cells were pre-treated with hBAFF protein. Then, WiL2-NS cells were added and co-incubated to adhere. It has been shown the data with different culture combination of MH7A and WiL2-NS cells (Supplementary Figure S3). When only MH7A cells were pre-treated with hBAFF (20 ng/mL), B cell adhesion to FLS was the highest rate compared to other conditions. The percentage of WiL2-NS cells bound to MH7A cells was 2.6 times greater than that in the control (Figure 1H). Taken together, these results suggest that B cell adhesion to FLSs is up-regulated by hVCAM1 expression via TNF-α-mediated hBAFF production.

### 2.2. hVCAM1 Expression Is Up-Regulated by TNF-α in MH7A Cells

**Figure 2:**
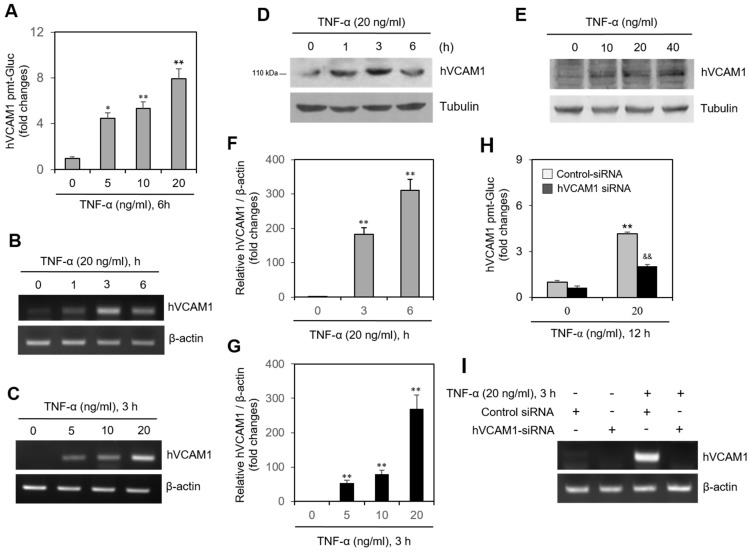
TNF-α increased human vascular cell adhesion molecule-1 (hVCAM1) expression. (**A**) MH7A cells were transfected with pEZx-PG02-hVCAM1-gaussia luciferase (Gluc) plasmid DNA using polyethylenimine (PEI), and treated with various concentrations of TNF-α. Gluc activity was measured by using luminometer. (**B**–**G**) MH7A cells treated with 20 ng/mL TNF-α for various times (**B**,**D**,**F**). hVCAM1 expression was detected by RT-PCR (**B**,**C**) or real-time Q-PCR (**F**,**G**). Cell lysates were prepared and hVCAM1 protein level was detected by western blot analysis (**D**,**E**). (**H**,**I**) MH7A cells were transfected with control or hVCAM1-siRNA to knockdown hVCAM1 expression by using Lipofectamine^®^. Then, cells were transfected with pEZx-PG02-hVCAM1-Gluc plasmid DNA by using PEI, and treated with 20 ng/mL TNF-α. Gluc activity was measured by using luminometer (**H**). RNA was purified with Nucleozol^®^. hVCAM1 expression was detected by RT-PCR (**I**). Each experiment was performed at least four times. Data in a bar graphs represent the means ± SD. * *p* < 0.05, ** *p* < 0.01; significantly different from TNF-α-untreated (**A**,**F**,**G**). or control siRNA-treated and TNF-α-untreated (**H**) control group. ^&&^ *p* < 0.01; significantly different from control siRNA-treated and TNF-α-treated control group (**H**).

In MH7A cells transfected with the pEZx-PG02-hVCAM1-gaussia luciferase (Gluc) plasmid, Gluc activity increased to 4.5, 5.5, and 7.9 times the original activity after treatment with various concentrations of TNF-α (Figure 2A). Treatment with TNF-α also increased the transcription level of hVCAM1, as detected by real-time reverse transcription polymerase chain reaction (RT-PCR), in a time-dependent (Figure 2B) and dose-dependent (Figure 2C) manner. Similarly, TNF-α treatment increased the protein level of hVCAM1, as detected by western blot analysis, in a time-dependent (Figure 2D) and dose-dependent (Figure 2E) manner. This time-dependent (Figure 2F) and dose-dependent (Figure 2G) increase in TNF-α-induced hVCAM1 expression was further confirmed by quantitative PCR (qPCR). The increase in Gluc activity of the TNF-α-treated MH7A cells transfected with pEZX-PG02-hVCAM1-Gluc plasmid was silenced ~50% by hVCAM1-siRNA (Figure 2H), as was the TNF-α-induced transcription level of hVCAM1 (Figure 2I). These data suggest that TNF-α up-regulates the transcriptional and translational levels of hVCAM1.

### 2.3. hVCAM1 Expression Is Associated with hBAFF Expression in MH7A Cells

**Figure 3:**
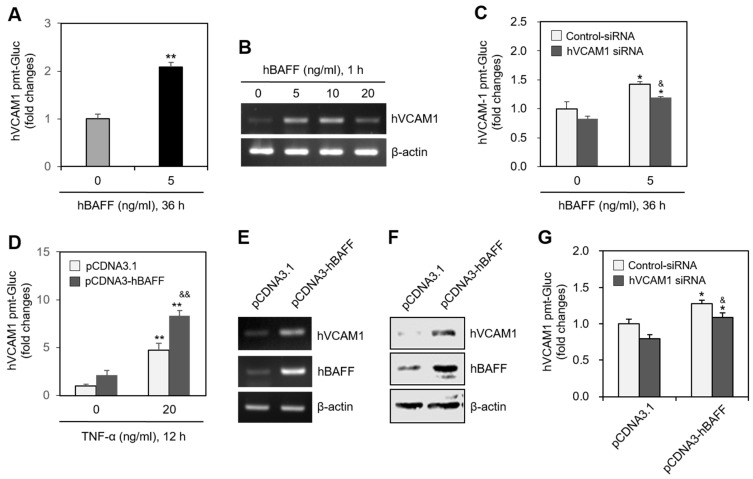
Human vascular cell adhesion molecule-1 (hVCAM1) expression was up-regulated by the treatment or the overexpression of hBAFF. (**A**) MH7A cells were transfected with pEZx-PG02-hVCAM1-gaussia luciferase (Gluc) plasmid DNA by using polyethylenimine (PEI), and treated with 5 ng/mL hBAFF protein. Gluc activity was measured by using luminometer. (**B**) MH7A cells were treated with various concentrations of hBAFF protein. RNA was purified with Nucleozol^®^. hVCAM1 expression was detected by RT-PCR. (**C**) MH7A cells were transfected with control or hVCAM1-siRNA to knockdown hVCAM1 expression by using Lipofectamine^®^. Then, cells were transfected with pEZx-PG02-hVCAM1-Gluc plasmid DNA by using PEI and treated with 5 ng/mL hBAFF protein. Gluc activity was measured by using luminometer. (**D**–**F**) MH7A cells were co-transfected with pCDNA3.1, or pCDNA3-hBAFF and pEZx-PG02-hVCAM1-Gluc plasmids by using PEI. Gluc activity was measured by using luminometer (**D**). RNA was purified with Nucleozol^®^. hVCAM1 expression was detected by RT-PCR (**E**). Cell lysates were prepared and hVCAM1 protein level was detected by western blot analysis (**F**). (**G**) MH7A cells were transfected with control or hVCAM1-siRNA to knockdown hVCAM1 expression by using Lipofectamine^®^. Then, cells were co-transfected with pCDNA3.1, or pCDNA3-hBAFF and pEZx-PG02-hVCAM1-Gluc plasmid DNA by using PEI. Gluc activity was measured by using luminometer. Each experiment was performed at least four times. Data in a bar graph represent the means ± SD. * *p* < 0.05, ** *p* < 0.01; significantly different from hBAFF protein-untreated (**A**,**C**) or pCDNA3.1-treated and TNF-α-untreated (**D**,**G**) or control group. ^&^ *p* < 0.05, ^&&^ *p* < 0.01; significantly different from control-siRNA-treated and hBAFF protein-treated (**C**) or pCDNA3.1-transfected and TNF-α-treated (**D**) or control-siRNA-treated and pCDNA3.1-hBAFF-transfected (**G**) control group.

**Figure 4:**
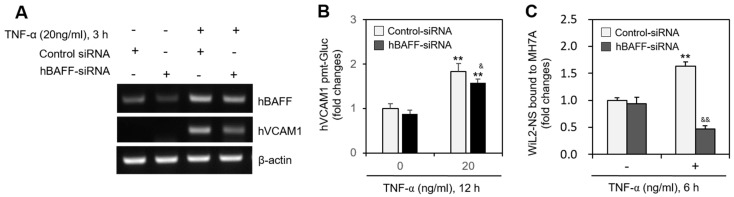
Human vascular cell adhesion molecule-1 (hVCAM1) expression was down-regulated by hBAFF-siRNA. (**A**–**C**) MH7A cells were transfected with control or hBAFF-siRNA to knockdown hBAFF expression by using Lipofectamine^®^. Cells were treated with 20 ng/mL TNF-α. RNA was purified with Nucleozol^®^. hVCAM1 and hBAFF expression was detected by RT-PCR (**A**). Cells were transfected with pEZx-PG02-hVCAM1 gaussia luciferase (Gluc) plasmid DNA by using PEI, and treated with 20 ng/mL TNF-α. Gluc activity was measured by using luminometer (**B**). Cells were treated with 20 ng/mL TNF-α for 6 h. Then, WiL2-NS cells were added and co-incubated to adhere for 30 min. Unbound WiL2-NS B cells were washed out and MTT assay was used to analyze bound B cells (**C**). Each experiment was performed at least four times. Data in a bar graph represent the means ± SD. ** *p* < 0.01; significantly different from control-siRNA-treated and TNF-α-untreated control group. ^&^ *p* < 0.01, ^&&^ *p* < 0.01; significantly different from control-siRNA-treated and TNF-α-treated control group (**A**,**C**).

In MH7A cells transfected with the pEZX-PG02-hVCAM1-Gluc plasmid, Gluc activity approximately doubled in response to 5 ng/mL hBAFF (Figure 3A), and hVCAM1 expression was also enhanced by 5 or 10 ng/mL hBAFF (Figure 3B). In MH7A cells transfected with hVCAM1-siRNA, the silenced hVCAM1 promoter activity increased in response to 5 ng/mL hBAFF (Figure 3C). To confirm the effect of hBAFF on hVCAM1 expression, MH7A cells were co-transfected with the pEZX-PG02-hVCAM1-Gluc and pcDNA3-hBAFF plasmids. Gluc activities in the hBAFF-overexpressed and TNF-α-treated groups were, respectively, 2.1 and 4.8 times greater than those in the control group (Figure 3D). The TNF-α-induced Gluc activity was 1.8 times greater in the hBAFF-overexpressed cells, compared with the pcDNA3.1 vector-transfected control. Thus, hVCAM1 was up-regulated, both transcriptionally and translationally, in the hBAFF-overexpressed cells, as determined by RT-PCR (Figure 3E) and western blot analysis (Figure 3F), respectively. When MH7A cells were co-transfected with pEZX-PG02-hVCAM1-Gluc and pcDNA3-hBAFF plasmid DNA in the presence of control or hVCAM1-siRNA, Gluc activity in the hVCAM1 siRNA-transfected group was inhibited ~18% compared with the control group (Figure 3G). In addition, when MH7A cells were transfected with hBAFF-siRNA and treated with TNF-α, hVCAM1 expression (Figure 4A) and hVCAM1 promoter activity (Figure 4B) decreased ~20%, compared with the control group. The TNF-α-induced increase in binding of WiL2-NS cells to MH7A cells was also inhibited ~75% in the group with hBAFF-siRNA compared with the control group (Figure 4C). These data suggest that hVCAM1 expression is regulated by hBAFF. It also suggests that B cell adhesion to FLSs is affected by TNF-α-mediated hBAFF production.

### 2.4. JNK Phosphorylation Is Associated with hVCAM1 Expression

**Figure 5:**
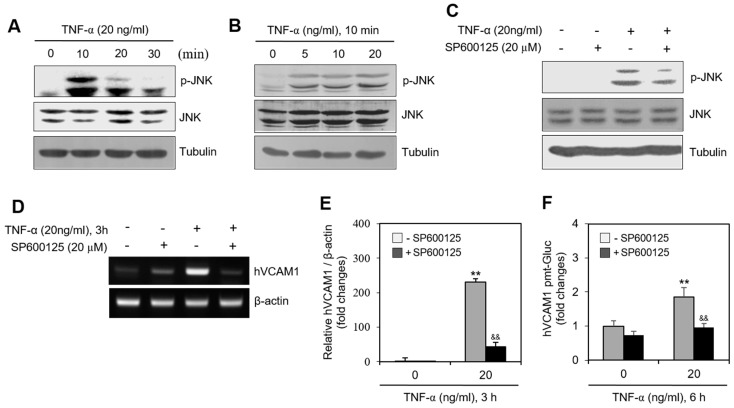
JNK activation by TNF-α regulated human vascular cell adhesion molecule-1 (hVCAM1) expression. (**A**,**B**) MH7A cells were treated with 20 ng/mL TNF-α for various times (**A**). MH7A cells were treated with various concentrations of 20 ng/mL TNF-α for 10 min (**B**). JNK and phosphorylated (p)-JNK were measured by western blot analysis. (**C**,**D**) MH7A cells were treated with 20 ng/mL TNF-α in the presence or absence of SP600125, JNK inhibitor. were used for inhibition of JNK activation and 20 μM of SP600125 had strong ability of inhibition of JNK activation. Cell lysates were prepared and protein level of JNK and p-JNK was detected by western blot analysis (**C**). RNA was purified with Nucleozol^®^. hVCAM1 and hBAFF expression was detected by RT-PCR (**D**). (**E**) MH7A cells were transfected with pEZx-PG02-hVCAM1-gaussia luciferase (Gluc) plasmid DNA by using polyethylenimine (PEI), and treated with TNF-α in the presence or absence of SP600125. Gluc activity was measured by using luminometer. (**F**) MH7A cells were treated with 20 ng/mL TNF-α for 6 h in the presence or absence of SP600125. Then, WiL2-NS cells were added and co-incubated to adhere for 30 min. Unbound WiL2-NS B cells were washed out and MTT assay was used to analyze bound B cells. Each experiment was performed at least four times. Data in a bar graph represent the means ± SD. ** *p* < 0.01; significantly different from SP600125-untreated and TNF-α-untreated control group. ^&&^ *p* < 0.01; significantly different from SP600125-untreated and TNF-α-treated control group (**E**,**F**).

To determine whether a kinase controls hVCAM1 expression, we assessed the levels of various signaling molecules. When MH7A cells were treated with TNF-α, JNK phosphorylation increased significantly for 10 min (Figure 5A) in a dose-dependent manner (Figure 5B). When MH7A cells were treated with TNF-α in the presence of SP600125, a JNK inhibitor both JNK phosphorylation (Figure 5C) and hVCAM1 expression (Figure 5D) were inhibited. The level of hVCAM1 expression in TNF-α-treated cells decreased ~80% in the presence of SP600125, as determined by qPCR (Figure 5E), while the hVCAM1 promoter activity in TNF-α-treated cells decreased ~50%, compared with the SP600125-untreated control (Figure 5F). These data suggest that hVCAM1 expression is regulated by activation of the JNK signaling pathway.

### 2.5. Interaction between MH7A Cells and WiL-2NS Cells Is Mediated by hVCAM1 Expression

**Figure 6:**
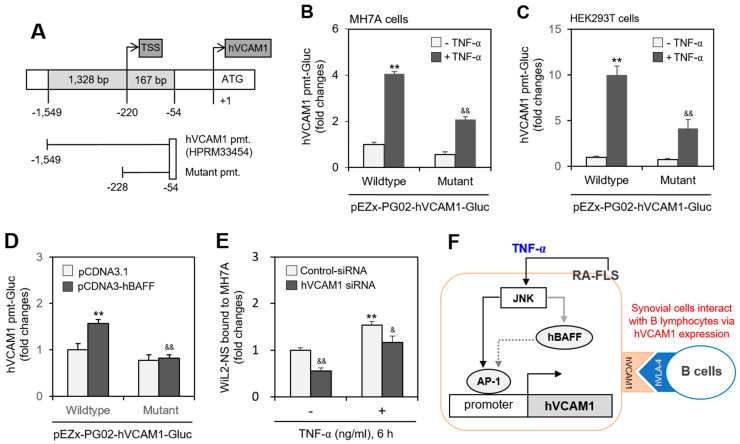
WiL2-NS cell bound to MH7A cells were enhanced by human vascular cell adhesion molecule-1 (hVCAM1) expression. (**A**–**C**) Mutant promoter to hVCAM1 was prepared from wild-type promoter (**A**). MH7A (**B**) or HEK293T (**C**) cells were transfected with wild- or mutant-type of pEZx-PG02-hVCAM gaussia luciferase (Gluc) plasmid DNA. Then, each cell was incubated for 30 h after transfection, and treated with 20 ng/mL TNF-α for additional 6 h. Gluc activity was measured by using luminometer (**B**,**C**). (**D**) MH7A cells were co-transfected with pCDNA3.1, or pCDNA3-hBAFF and wild or mutant type of pEZx-PG02-hVCAM-Gluc plasmid DNA by using PEI. Gluc activity was measured by using luminometer. (**E**) MH7A cells were transfected with control or hVCAM1-siRNA to knockdown hVCAM1 expression by using Lipofectamine^®^ and treated with 20 ng/mL TNF-α for 6 h. Then, WiL2-NS cells were added and co-incubated to adhere for 30 min. Unbound WiL2-NS B cells were washed out and MTT assay was used to analyze bound B cells. Each experiment was performed at least four times. Data in a bar graph represent the means ± SD. ** *p* < 0.01; significantly different from TNF-α-untreated (**B**,**C**) or pCDNA3.1-transfected (**D**) or control-siRNA-treated and TNF-α-untreated (**E**) control group. ^&^ *p* < 0.05, ^&&^ *p* < 0.01; significantly different from control TNF-α-treated (**B**,**C**) or pCDNA3-hBAFF-transfected (**D**) or control-siRNA-treated and TNF-α-treated (**E**) control group. (**F**) This is a schematic interaction mechanism of B lymphocytes to synovial cells by hVCAM1 expression under TNF-α stimulation. It suggests that TNF-α-activated JNK controls hVCAM1 expression via AP-1 binding on its promoter (black solid line) in synovial cells. JNK also might induce hBAFF expression (grey solid line), which lead to the regulation of hVCAM1 expression by AP-1 binding on its promoter (grey dotted line) in synovial cells. Through this molecular mechanism, hVCAM1 can control the interaction of synovial cells to B lymphocytes.

Since hVCAM1 expression was influenced by JNK activation (Figure 5), we investigated whether the hVCAM1 promoter is regulated by a JNK-associated factor. Analysis of the hVCAM1 promoter sequence against the TRANSFAC (version 8.3) database indicated a binding site for activator protein 1 (AP-1) in the 614~607-bp region (Supplementary Figure S1, online). To test the interaction between AP-1 and the hVCAM1 promoter, we prepared a deletion mutant (174 bp, −228~−54 region, upstream of the hVCAM1 promoter) of the hVCAM1 promoter (Figure 6A). When MH7A or human embryonic kidney (HEK293T) cells were transfected with wild-type or mutant pEZX-PG02-hVCAM1-Gluc plasmids and incubated in the presence of TNF-α, Gluc activity decreased ~50% or ~60%, respectively, compared with TNF-α-treated control cells (Figure 6B,C). To confirm the effect of hBAFF on the AP-1 binding site of the hVCAM1 promoter, MH7A cells were co-transfected with wild-type or mutant pEZX-PG02-hVCAM1-Gluc and pcDNA3-hBAFF plasmids. Wild-type Gluc activity in the hBAFF-overexpressed group was 1.6 times that of the control pcDNA3.1 vector-transfected group (Figure 6D). However, no increase in mutant-type Gluc activity was detected in groups with or without hBAFF overexpression using pcDNA3-hBAFF or pcDNA3.1 control plasmids. In addition, when MH7A cells were transfected with hVCAM1-siRNA and then treated with TNF-α, the percentage of WiL2-NS cells bound to MH7A cells decreased by ~50%, compared with the control group. The percentage of WiL2-NS cells bound to MH7A cells after TNF-α treatment was also diminished by ~25% by subsequent treatment with hVCAM1-siRNA, compared with control siRNA (Figure 6E). These data suggest that hVCAM1 mediates the interaction of FLSs with B lymphocytes via TNF-α-induced activation of the JNK signaling pathway. They also suggest that hVCAM is regulated by AP-1 binding on the hVCAM promoter via JNK-mediated hBAFF expression (Figure 6F).

## 3. Discussion

RA is an inflammatory disease that leads to progressive joint destructions. Not only hyperplastic rheumatoid synovium but also infiltration into cartilage and bone are mainly the phenomenon of RA [2,3,4,5]. Synovium is almost composed of fibroblast-like synoviocytes (FLSs) that interact with many immune cells such as T, B cells and macrophages [6,7]. FLSs produce cytokines such as TNF-α that is one of the most important cytokines in RA [17]. TNF-α upregulated the expression of BAFF [12,13,15] and hVCAM1 [12,14,27,30]. hVCAM1 interacts with its counter molecule, VLA-4 (integrin α4β1) on leukocytes, which led to recruit them into inflammation site [14,30,36]. However, little information has been known about the interaction between B cells and FLSs through BAFF-mediated VCAM1 expression. Thus, we investigated whether B cell interaction to FLSs could be associated with hVCAM1 expression via TNF-α-induced BAFF expression.

TNF-α treatment of MH7A cells, a synovial cell line derived from human RA patients, increased the binding affinity of the synovial cells to WiL2-NS B lymphocytes (Figure 1). TNF-α also augmented the expression of hBAFF and hVCAM1 (Figure 2). Overexpression or knockdown of hBAFF, by transfection with a pcDNA3-hBAFF plasmid or hBAFF-siRNA, respectively, confirmed that hBAFF may up-regulate hVCAM1 expression in MH7A cells (Figure 3 and Figure 4). TNF-α also appeared to promote activation of the JNK signaling pathway, leading to regulation of hVCAM1 expression (Figure 5). Deletion upstream of the hVCAM1 promoter reduced the transcriptional activity induced by TNF-α or by hBAFF overexpression. Silencing of hVCAM1 expression by hVCAM1-siRNA inhibited the cell-to-cell interaction of B lymphocytes with MH7A synoviocytes (Figure 6). Taken together, our data suggest that hBAFF regulates hVCAM1 expression, which in turn regulates cell-to-cell interactions.

Although BAFF is a TNF-α family protein, those bind to their own specific receptors. BCMA, TACI and BAFF receptor, BAFF-R are receptors for BAFF [12,13]. TNFR1 and TNFR2 are receptors for TNF-α [21,22,23]. Our data showed that TNF-α as the main cytokine increased the expression of BAFF and VCAM1. Then, we focused on hierarchical expression order of target genes stimulated by TNF-α, which could be associated with VCAM1 expression in synovial cells activated by TNF-α-mediated BAFF. Thus, it suggests VCAM1 could be expressed through TNF-α-JNK-BAFF axis (Figure 6F).

TNF-α can induce BAFF expression in RA FLSs via the c-Fos proto-oncogene and reactive-oxygen-species-dependent protein kinase C pathway [12,38]. TNF-α enhances JNK phosphorylation in synovial cells (Figure 5) [39], which is consistent with our conclusion that the hVCAM1 promoter includes an AP-1 binding site (Supplementary Figure S1). AP-1, a mammalian transcription factor, is not a single protein [40] and is involved in various cell processes through the regulation of nuclear gene expression [41]. It comprises four subfamilies: Fos (c-Fos, FosB, Fra1, and Fra2), Jun (c-Jun, JunB, and JunD), ATF-activating transcription factor (ATF2, LRF1/ATF3, BATF, JDP1, and JDP2), and musculoaponeurotic fibrosarcoma (c-Maf, MafB, MafA, Mafg/f/k, and Nrl). Given this complexity, further research is required to define which subfamily of AP-1 mediates hVCAM1 expression to enhance the interaction of B cells with FLSs.

Expression of hBAFF in FLSs has also been increased by hypoxia-induced factor 1α binding to the hBAFF promoter through extracellular signal-regulated kinase activation [42]. In addition, BAFF expression has been associated with the activation of NF-κB in lipopolysaccharide-stimulated mouse spleen cells [43,44], the Janus kinase-signal transducer and activator of transcription signaling pathway in interferon-γ-stimulated human intestinal epithelial cells [45], the CREB-binding protein/p300 [44], Epac1-mediated Rap1, and protein kinase A-mediated CREB in mouse macrophages [46,47]. We suspect that a wide variety of molecules that regulate BAFF expression may also affect hVCAM expression, leading to an increase of B cell interaction with FLSs.

MH7A cells are FLS immortalized by the transfection with the SV40 T antigen [48] and then treated with TNF-α to increase its binding affinity to B cell. Our data showed that TNF-α treatment increased the binding affinity of the synovial cells to Wil2-NS B lymphocytes (Figure 1G). In addition, the adhesion of B cells to FLS through VCAM-1 contributes to the survival of B lymphocytes at the site of inflammation in RA [37]. VCAM1 was expressed on cell surface of RA-FLSs, osteoarthritis (OA)-FLSs, or IL-4-stimulated dermal fibroblasts but not on non-stimulated dermal fibroblasts. Mature B-cell migration beneath dermal fibroblasts is significantly enhanced by IL-4 treatment of dermal fibroblasts, which is significantly inhibited by anti-CD106 monoclonal antibodies [47]. So, the binding affinity of FLS to B cell adhesion could be also differently regulated by the level of VCAM1.

FLSs also play a role as ‘nurse-like’ cells to mature B cells [49]. A few possibilities explain why the passage has bound over the adhesion of WiL2-NS cells with MH7A cells (Figure 1E). As the passage increases, the first is that nurse-like function of FLS might be enhanced to support B cells [47]. The second is that senescence in FLSs could lead to one life-saving or another detrimental outcome [50] such as joint inflammation by the increased adhesion of B cells. Synoviocyte activation induces the maturation of B cells into plasma cells [51]. B cell adhesion to FLSs contributes to B cell survival at the inflammation site [37]. Therefore, interactions of FLSs with B cells could be a critical process in RA. Synovial cells express not only VCAM1 but also various cell surface molecules, including chemokines, adhesion molecules, and cytokines, to modulate B lymphocytes [52]. These molecules may also contribute to the interaction of FLSs with B cells through BAFF-mediated expression.

In conclusion, exposure to TNF-α enhances hVCAM1 expression in FLSs via hBAFF expression and JNK activation. The increased level of hVCAM1 expression contributed to the increase of B cell binding to FLSs. Thus, interactions between B lymphocytes and synovial cells may be regulated by hBAFF-mediated hVCAM1 expression via activation of the JNK signaling pathway. Although the mechanism by which hBAFF regulates hVCAM1 expression is unknown, TNF-α-induced hVCAM1 expression appears to promote interactions between WiL2-NS B cells and MH7A synovial cells. Controlling the interactions between B lymphocytes and synovial cells may be an efficient therapeutic strategy to alleviate RA.

## 4. Material and Methods

### 4.1. Reagents

Recombinant human TNF-α was obtained from R&D System Inc. (Minneapolis, MN, USA). Antibodies to phospho-JNK (Thr 183/Tyr 185) and JNK were purchased from Cell signaling Technology (Beverly, MA, USA). Antibodies to hVCAM1 were purchased from Santa Cruz Biotechnology, Inc. (Santa Cruz, CA, USA). Anti-BAFF antibodies (PRS2221) and MTT [3(4,5-dimethyl-thiazol-2-yl)-2,5-diphenyl tetrazolium bromide] (M5655) were purchased from Sigma-Aldrich (St. Louis, MO, USA). Small interference(si) RNAs are customer-ordered to Bioneer (Daejeon, Korea). Sequences for each siRNA were as follows; hBAFF-siRNA-1 (sense; CU CCA ACU UGC AAU ACC AA, anti-sense; UU GGU AUU GCA AGU UGG AG), hBAFF-siRNA-2(sense; UG AAG GAG UGU GUU UCC AU, anti-sense; AU GGA AAC ACA CUC CUU CA), hVCAM1-siRNA (sense; AA UGC AAC UCU CAC CUU AA, anti-sense; UU AAG GUG AGA GUU GCA UU). AccuTarget™ negative control siRNA (SN-1001) was also purchased from Bioneer (Daejeon, Korea).

### 4.2. Collection of Human RA-FLS

Informed consent was obtained from all patients, and the experimental protocol was approved by the Konkuk University Medical Center Institutional Review Board. RA patients fulfilled the criteria of the American College of Rheumatology (formerly, the American Rheumatism Association) [53]. RA FLS were isolated from the synovial tissues according to a protocol as follows. Briefly, synovial tissues were washed thoroughly with RPMI 1640 (Gibco BRL, Gaithersburg, MD, USA), minced into 1 mm^3^, and digested for 90 min. at 37 °C in RPMI 1640 containing 1 mg/mL collagenase (Gibco BRL). The digested tissue was filtered with a 70 μm cell strainer (Becton Dickinson, Franklin Lakes, NJ, USA), and centrifuged at 250× *g* for 10 min. The cell pellet was resuspended in RPMI 1640, washed 3 times by centrifugation, and suspended in α-minimum essential medium (α-MEM; Irvine Scientific, Santa Ana, CA, USA) containing 10% fetal bovine serum (FBS) (Gibco BRL). The cells were then subcultured for 3–6 passages before use.

### 4.3. Cell Cultures

WiL2-NS, a human B lymphoblast cells was acquired from the Korea Research Institute of Bioscience and Biotechnology (KRIBB) cell bank (Taejeon, Korea). MH7A synovial cells isolated from intra-articular soft tissues of the knee joints of RA patients were obtained from the Riken cell bank (Ibaraki, Japan) through Dr. Ho-Geun Yoon of Yonsei University (Seoul, Korea). MH7A is a cell line established by transfection with the SV40 T antigen [48]. Briefly, both WiL2-NS and MH7A cells were cultured in RPMI-1640 (Gibco BRL, Gaithersburg, MD, USA) supplemented with 10% heat-inactivated fetal bovine serum (FBS), penicillin (final concentration, 100 U/mL), streptomycin (final concentration, 0.1 mg/mL) and 2 mM L-glutamine (GIBCO, Grand Island, NY, USA) at 37 °C humidified incubator in an atmosphere of 5% CO_2_ in air [42,54].

### 4.4. Quantitation of the Interaction of MH7A with WiL2-NS Cells

The interaction of MH7A with WiL2-NS cells was measured as follows. Briefly, 1 × 10^5^ number of MH7A cells were plated on 24-well plate 1 day before the experiments. After, 1 × 10^5^ number of WiL2-NS cells were added to MH7A cells and co-incubated for 30 min. Then, a 24-well plate was gently tapped twice to remove unbound WiL2-NS cells by taking the medium out and washing each well with PBS. Cell density was measured by MTT assay [55]. MTT (300 µg/mL) solution was added and incubated for an additional 1–2 h until insoluble purple formazan is visible. Supernatant medium was discarded from each well and formazan formed by MTT were dissolved by using 400 µL dimethylsulfoxide (DMSO). Then, optical density (OD) was measured at 540 nm. WiL2-NS cells bound to MH7A cells were calculated by the subtraction of OD with MH7A cells from OD with co-incubation of both cells.

### 4.5. Transfection of Nucleic Acids

Each plasmid DNA, siRNAs for hVCAM1 or hBAFF and AccuTarget™ negative control siRNA were transfected into cells as follows [54]. Briefly, each nucleic acid and lipofectamine 2000^®^ (Invitrogen, Calsbad, CA, USA) was diluted in serum-free medium and incubated for 5 min, respectively. The diluted nucleic acid and lipofectamine 2000^®^ reagent was mixed by inverting and incubated for 20 min to form complexes. In the meantime, cells were stabilized by the incubation with culture medium without antibiotics and FBS for at least 2 h prior to the transfection. Pre-formed complexes were added directly to the cells and cells were incubated for an additional 6 h. Then, culture medium was replaced with antibiotic and 10% FBS-containing DMEM and cells were incubated at 37 °C in CO_2_ incubator for 24–48 h prior to each experiment [56].

### 4.6. Gaussia Luciferase Assay

Pre-designed promoters for hVCAM1 (NM_001078) were obtained from GeneCopoeia Inc. (Rockville, MD, USA). hVCAM1-promoter (HPRM33454) was 1328 bp (−1549~−54) upstream from starting codon for hVCAM1 transcription in Homo sapiens BAC RP11-86F24 from chromosome 1 (AC093428.2). Schematic figures and sequences of promoters were shown in Supplementary Figure S1. hVCAM1 mutant promoter 174 bp (−228~−54) was prepared by the deletion of wildtype upstream. Wildtype and mutant promoters were cloned into Gaussia luciferase (Gluc) reporter plasmid vector, pEZX-PG02.

MH7A cells were transfected with wildtype or mutant pEZX-PG02-hVCAM1-Gluc plasmid DNA using PEI (Invitrogen, Calsbad, CA, USA) to measure the activity of hVCAM1-promoter. At the same time, cells were co-transfected with pcDNA-lacZ for monitoring transfection efficiency by β-galactosidase assay. Then, cells were incubated for an appropriate time. Secreted Gluc reporter protein was obtained by the collection of culture-conditioned media after the indicated time intervals. Gluc activity of reporter protein was measured by BioLux^®^ Gluc assay kit (New England BioLabs, Ipswich, MA, USA) including coelenterazine as a substrate for Gluc according to the manufacturer’s protocol. Luminescence was measured using luminometer (Berthold Technologies, Oak Ridge, TN, USA). Luciferase activity unit was normalized to this control β-galactosidase activity [56].

### 4.7. Reverse Transcriptase Polymerase Chain Reaction (RT-PCR)

Total RNA was extracted from MH7A cells using Nucleozol^®^ (BMS). cDNA was synthesized from 1ug of total RNA using oligo-dT_18_ primers (Macrogen, Seoul, Korea) and superscript reverse transcriptase (Bioneer, Daejeon, Korea) in a total volume of 21 uL. For standard PCR, 1 µL of the first-strand cDNA product was then used as a template for PCR amplification with Taq DNA polymerase (Cosmo Genetech, Seoul, Korea). PCR amplification was performed using 10 pmol of specific primers specific for human BAFF (forward; aat tca gag gaa ggt cc, reverse; atg tga cat ctc cat cca gt), VCAM1(forward; gga acg aac act ctt acc t, reverse; gca act gaa cac ttg act g), integrin β1 (forward; cag cag ttg gtt ttg cga tt, reverse; atg cgc tgt ttt cca aca ag) and β-actin (forward;gtc acc aac tgg gac gac at, reverse; gca cag cct gga tag caa cg) with 36 thermocycles (95 °C for 40 s, 57 °C for 30 s and 72 °C for 60 s). PCR products were detected by agarose gel electrophoresis.

### 4.8. Quantitative Real-Time RT-PCR

To perform real-time quantitative PCR (qPCR), total cellular RNA (5 μg) was reverse transcribed into cDNA as described in RT-PCR [54]. Real-time qPCR was performed using the CFX96 Touch™ Real-Time PCR Detection System (Bio-Rad laboratories, Hercules, CA, USA). Relative transcripts levels were measured in 20 µL reactive volume. The RT reaction product (100 ng) was amplified with THUNDERBIRD™ SYBR qPCR mix (TOYOBO Co. Ltd., Osaka, Japan) using primers specific for target genes, hVCAM1 (forward; gga acg aac act ctt acc t, reverse; gca act gaa cac ttg act g) and β-actin (forward; gcc agg tca tca cca ttg, reverse; gtt gaa ggt agt ttc gtg gat), with 40 thermocycles (95 °C for 10 s, 55 °C for 10 s and 72 °C for 30 s). Relative quantification of hVCAM1 mRNA was analyzed by the comparative threshold cycle (CT) method and normalized to β-actin expression using Bio-Rad CFX Manager™ Software.

### 4.9. Western Blot Analysis

Whole cells were lysed with lysis buffer which contained 0.5% Nonidet P-40 (*vol/vol*) in 20 mM Tris-HCl (pH 8.3); 150 mM NaCl; protease inhibitors (2 μg/mL) aprotinin, pepstatin, and chymostatin; 1 μg/mL leupeptin and pepstatin; 1 mM phenylmethyl sulfonyl fluoride (PMSF); and 1 mM Na4VO3. Lysates were incubated for 1 h on ice before centrifugation at 13,000 rpm for 20 min at 4 °C. Proteins were measured using a bicinchoninic acid (BCA) protein assay. dye reagent and denatured with boiling for 5 min in sodium dodecyl sulfate (SDS). Sample buffer Proteins were separated by SDS-polyacrylamide gel electrophoresis (SDS-PAGE) and transferred to nitrocellulose membranes by electroblotting. After the membranes were blocked in 5% skim milk in Tris-buffered saline with Tween 20 (TBST; 10 mM Tris-HCl, pH 7.6; 150 mM NaCl; 0.5% Tween 20) and incubated with the indicated antibodies. The membrane was washed twice with TBST and incubated with antibodies against hVCAM1 (1:100) and tubulin (1:5000). Horseradish peroxidase (HRP)-labeled secondary anti-mouse or anti-rabbit antibodies were diluted 1:5000 in TBST containing 0.5% Tween 20. Antibodies were detected with HRP-conjugated secondary antibodies with the use of enhanced chemiluminescence (ECL; Pierce, Rockford, IL, USA). Immunoreacted bands were detected using X-ray film [42].

### 4.10. Statistical Analysis

Statistical significance for experimental differences was tested using ANOVA and student’s *t*-test. *p* values of <0.05 or <0.01 were considered to indicate significance.

## Supplementary Materials

The following are available online at https://www.mdpi.com/article/10.3390/ijms22137166/s1.
