# Supplementary material for: B Cell Adhesion to Fibroblast-Like Synoviocytes Is Up-Regulated by Tumor Necrosis Factor-Alpha via Expression of Human Vascular Cell Adhesion Molecule-1 Mediated by B Cell-Activating Factor
Source: Int J Mol Sci. 2021 Jul 2;22(13):7166. doi: 10.3390/ijms22137166 (PMC8267633; doi:10.3390/ijms22137166)
Supplement: Supplementary file 1 [file ijms-22-07166-s001.zip › ijms-1229046-supplementary.pdf]

B cell adhesion to fibroblast-like synoviocytes is up-regulated by tumor necrosis factor-alpha via expression of human vascular cell adhesion molecule-1 mediated by B cell-activating factor

Sung-Sik Yoon and Eun-Yi Moon*

Department of Bioscience and Biotechnology, Sejong University, Seoul 05006, Republic of Korea

Running title: Up-regulation of B cell adhesion to FLS by hVCAM-1 expression

*Corresponding author
Eun-Yi Moon, Department of Bioscience and Biotechnology, Sejong University, 209 Neungdong-ro Kwangjin-gu, Seoul 05006, Republic of Korea,
Tel: +82 2 3408 3768; Fax: +82 2 466 8768.
E-mail address: eunyimoon@sejong.ac.kr (E.Y. Moon)

A.

hVCAM1 promotor (HPRM33454)

Length 1,496 bp

-1,539 ~ -54 from CDS

-1,328 ~ +167 from TSS

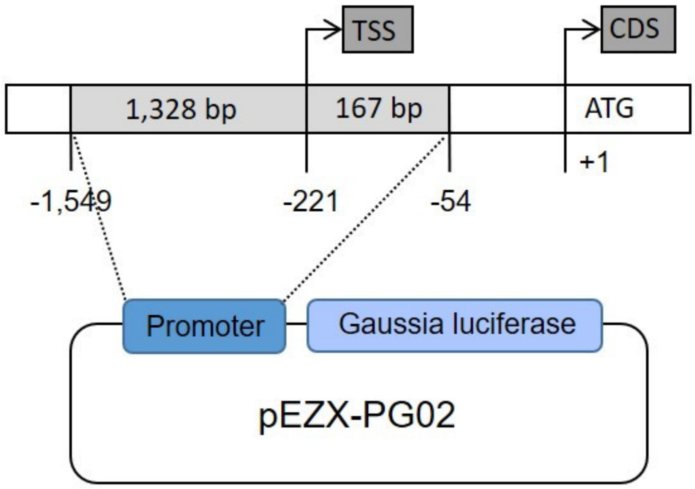

B.

-1,549 tccccacac cctagaacag catctccaac ttatttttcc ctccttgtct
-1,500 tttagtggga gccacatcag tatccaagag gagatccaga agcctctcca accaggtagg
-1,440 gacagttata gattccagac ctcagctatg gcctttgtta cagagtacaa atgttatata
-1,380 gtacaagttt attgtacaca tcccattgag tctctgagct ttagaatttt cttgtagaat
-1,320 ttaacagttt tttcatgccg tatttacata ttattgctag tatttagaat tttcttctcc
-1,260 aaatgtataa cgtttattat tgcatttttt gtatccacta agtggaaaat catgcattag
-1,200 atattgtaga agtagataca acaatgaaca agaactggtc ctgaccatga gaggaactga
-1,140 tgatccaatg ggggagatag acctgcacgt gtttaataaa aggaagtggc tattccggtt
-1,080 tctttttgat gggcaagcat tttgcaaggc cttgggctat gtgtgtgcaa ggctaagcca
-1,020 gttagttaat tgggattttt ttaaaaaggc acttcactgg ggggaaaagg aacatagagt
-960 tggttattgt ccccttgcct ataataaaaa cctattattt ttaatttttt aactgggttt
-900 gcggttaaat ctcacagccc aagagatttg ccacttcaga tggattccat acacttgcat
-840 ttaagtatgc aaaaaaattc caattatcca gcaatttaac caaattattg gtaacttttc
-780 taaaacaaaa aaaaattgtt tcccttgttt tggcagcaat ttcagttaca gtcctttact
-720 ttctactcaa gaaaatagtt tcaaaaagtt gatgtttgtt gctaaaagaa ctatttttat
-660 gaataaatat aaaactaaga agttatggtg tccctttttt aaaaaa**tgac** **tcat**caaaag
-600 aaataacttt ttcctttctc ttgtaagaga aaaaaattaa tctcttttag aattgcaaac
-540 atatttcctt gatggagaaa atcaattcac atggcatagt cgttatttat ccagttcaaa
-480 aaccagagta gaatttacta ctctgtctcc atttttctc tccccacccc cttaacccac
-420 attggattca gaaagcttca ttctgcaatc agcattgtcc tttatctttc cagtaaagat
-360 agccttttgg agtcgaagat gaggaaaagc ctgtatttta tagtcttgga agtgtcttct
-300 tttgccagga cagagagagg agcttcagca gtgagagcaa ctgaaggggt taatagtgga
-240 acttggctgg gtgtctgtt**a** aactttttc cctggctctg ccctgggttt cccctttgaag
-180 ggatttccct ccgcctctgc aacaagaccc tttataaagc acagactttc tatttcactc
-120 cgcggtatct gcatcgggcc tcactggctt caggagctga ataccctccc aggcacacac
-60 aggtggg*aca caaataaggg ttttggaacc actattttct catcacgaca gcaacttaaa*
+1 ***atg****cctggga agatggtcgt gatccttgga gcctcaaata tactttggat aatgttt*

Supplementary Figure S1. Schematic figures and sequences of pre-designed hVCAM1 (NM_001078) promoter. (A) hVCAMpromoter (HPRM33454) was 1,328 bp (-1,549 ~ -54) upstream from starting codon, ATG, of coding sequence (CDS) for hVCAM1 transcription. hVCAM1 promoter covers 1,328 bp upstream and 167 bp downstream from transcription starting site (TSS). (B) Sequence of hVCAM1 promoter includes AP-1 binding sites on -614 ~ -607 (black bold underlined) which is predicted by using TRANSFEC (version 8.3) database, TSS (black bold), and translation starting codon (italic black bold underlined) which is shown in between -53 and 57 bp (italic grey bold underlined).

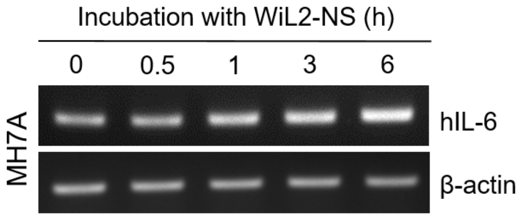

Supplementary Figure S2. IL-6 expression in MH7A cells interacted with WiL2-NS cells. MH7A cells were plated overnight. Then, WiL2-NS cells were added and co-incubated to adhere for 0.5, 1, 3 and 6 h. WiL2-NS cells were separated and MH7A cells were collected at each time point. RNA was purified with Nucleozol®. The expression of hIL-6 in MH7A cells was respectively detected by RT-PCR.

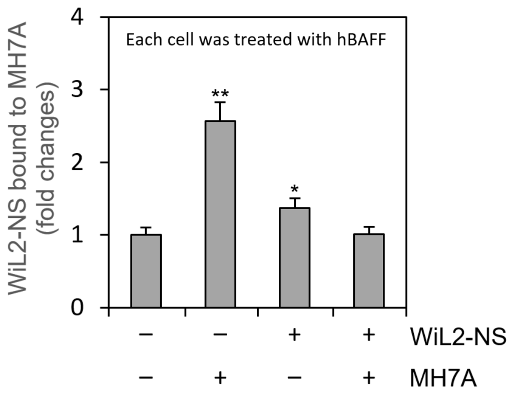

Supplementary Figure S3. Interaction of MH7A cells with WiL2-NS cells was increased by the pre-treatment with hBAFF protein. MH7A cells were plated overnight. MH7A and WiL2-NS cells were pre-treated with hBAFF (20 ng/mL) for 30 min. Then, WiL2-NS cells were added and co-incubated to adhere for 30 min. Unbound WiL2-NS B cells were washed out and MTT assay was used to analyze bound B cells. Experiment was performed at least four times. Data in a bar graph represent the means ± SD. *p<0.05, **p<0.01; significantly different from hBAFF protein-untreated control group.
